# Hypothermia Promotes Interleukin-22 Expression and Fine-Tunes Its Biological Activity

**DOI:** 10.3389/fimmu.2017.00742

**Published:** 2017-06-29

**Authors:** Evgeny Chichelnitskiy, Britta Himmelseher, Malte Bachmann, Josef Pfeilschifter, Heiko Mühl

**Affiliations:** ^1^Pharmazentrum Frankfurt/ZAFES, University Hospital Goethe-University Frankfurt, Frankfurt, Germany

**Keywords:** interleukin-22, signal transducer and activator of transcription 3, endotoxemia, hypothermia, peripheral blood mononuclear cells

## Abstract

Disturbed homeostasis as a result of tissue stress can provoke leukocyte responses enabling recovery. Since mild hypothermia displays specific clinically relevant tissue-protective properties and interleukin (IL)-22 promotes healing at host/environment interfaces, effects of lowered ambient temperature on IL-22 were studied. We demonstrate that a 5-h exposure of endotoxemic mice to 4°C reduces body temperature by 5.0° and enhances splenic and colonic *il22* gene expression. In contrast, tumor necrosis factor-α and IL-17A were not increased. *In vivo* data on IL-22 were corroborated using murine splenocytes and human peripheral blood mononuclear cells (PBMC) cultured upon 33°C and polyclonal T cell activation. Upregulation by mild hypothermia of largely T-cell-derived IL-22 in PBMC required monocytes and associated with enhanced nuclear T-cell nuclear factor of activated T cells (NFAT)-c2. Notably, NFAT antagonism by cyclosporin A or FK506 impaired IL-22 upregulation at normothermia and entirely prevented its enhanced expression upon hypothermic culture conditions. Data suggest that intact NFAT signaling is required for efficient IL-22 induction upon normothermic and hypothermic conditions. Hypothermia furthermore boosted early signal transducer and activator of transcription 3 activation by IL-22 and shaped downstream gene expression in epithelial-like cells. Altogether, data indicate that hypothermia supports and fine-tunes IL-22 production/action, which may contribute to regulatory properties of low ambient temperature.

## Introduction

Interleukin (IL)-22 is a member of the IL-10 cytokine family that, predominantly by engaging signal transducer and activator of transcription (STAT)-3 signaling, modulates gene expression foremost in epithelial (-like) cells (1). A hallmark of IL-22 activity is pro-proliferative and anti-apoptotic action that combines with antimicrobial properties (2–4). IL-22 is, due to restricted expression of the IL-22 receptor chain-1, generally unable to target leukocytes (5). Since IL-22 fails to efficiently activate nuclear factor-κB (6), this cytokine displays a unique tissue-protective quality in absence of a direct effect on immunoactivation, neither in proinflammatory nor in immunosuppressive sense. Those favorable properties concur with protection by IL-22 as detected in rodent models of infection- and/or tissue damage-driven diseases at host/environment interfaces (4), which include intestinal *Citrobacter rodentium* (7) as well as lung *Klebsiella* pneumonia (8) and influenza infections (9), ventilator-induced lung injury (10), experimental colitis (11, 12), and acute liver injury (13, 14). In addition, by strengthening the crucial parameter of intestinal barrier integrity (15), local IL-22 is supposed to prevent translocation of pathogenic bacteria that otherwise pose a risk for sepsis development (16). This function is also supported by superior intestinal wound healing under the influence of IL-22 (12).

Most relevant sources of IL-22 are lymphoid cells, in particular group 3 innate lymphoid cells, NKT cells, γδT cells as well as differentiated Th1, Th17, and Th22 cells (2). Research aiming at characterizing molecular mechanisms driving *IL22* transcription has primarily focused on transcription factors that relate to lymphoid cell differentiation. For instance, STAT3, B-cell-activating transcription (Batf), retinoid orphan receptor γτ, and ligand-activated aryl hydrocarbon receptor all connect to Th17 differentiation, can bind the *IL22* promoter, and accordingly promote IL-22 production (17). Notably, information on transcription factors enabling instant *IL22* gene expression after T cell receptor activation is scarce. It is noteworthy that binding of NFATc2 to the *IL22* promoter has been linked to rapid (within 1 h) cyclosporin A (CsA)-sensitive induction of IL-22 mRNA in activated human Jurkat T cells (18).

Therapeutic hypothermia, frequently associated with diminished inflammation, is employed or recommended for selected clinical conditions, among others, cardiac surgery and traumatic brain injury (19, 20). Interestingly, upregulation of tissue-protective IL-22 has been observed in experimental traumatic brain injury and in patients undergoing cardiac surgery (21, 22). Basic science revealed that, in similarity to IL-22 (10), hypothermia ameliorates tissue injury in rat ventilator-induced lung injury (23). Moreover, exposure of mice to low ambient temperature, like IL-22 (13, 14), reduces acute liver injury (24). Interestingly, upregulation of IL-22-related anti-inflammatory IL-10 (25) associates with hypothermia in the context of experimental ventilator-induced lung injury (26, 27), severe trauma by fracture and hemorrhage (28), cardiac surgery (29), and systemic inflammatory response syndrome/endotoxemia (30–35). Given the tissue-protective properties of IL-22 (4), it is an important topic of current research to understand and develop strategies that aim at controlled upregulation of IL-22, especially during acute injury. To assess a potential link between hypothermia, tissue-protective responses, and IL-22 during inflammation/immunoactivation, we set out to investigate IL-22 in the context of lowered ambient temperature.

## Materials and Methods

### Reagents

Endotoxin (lipopolysaccharide, LPS, O55:B5) and brefeldin A were from Sigma-Aldrich (Taufkirchen, Germany). 12-O-tetradecanoylphorbol-13-acetate (TPA) was from Enzo Life Sciences (LÖrrach, Germany) and A23187 from AppliChem (Karlsruhe, Germany). Cyclosporin A (CsA) and FK506 were purchased from Calbiochem-Novabiochem (Bad Soden, Germany). Human IL-22 and interferon (IFN)γ were obtained from Peprotech Inc. (Frankfurt, Germany). Murine (αCD3-#17A2, αCD28-#37.51) and human (αCD3-#OKT3, αCD28-#28.2) agonistic anti-CD3 and anti-CD28 antibodies were from BioLegend (San Diego, CA, USA).

### *In Vivo* Mouse Experiments

All animal procedures were approved by local authorities (“Regierungspräsidium Darmstadt”) and are in accordance with National Institutes of Health guidelines. For experiments, 10- to 12-week-old C57Bl/6 male mice (MFD-Diagnostics GmbH, Wendelsheim, Germany) were transferred individually into cages early morning. The body weight and core temperature was determined using laboratory scales and a TH-5 + RET-3 mouse thermometer with rectal probes (Physitemp Instruments Inc., Clifton, NJ, USA). Mice were injected i.p. with LPS (1 µg/g body weight) or PBS and kept at either standard room temperature (RT, 23°C) or at 4°C with access to water only (36). After 5 h, mice underwent short isoflurane (Abbott, Wiesbaden, Germany) anesthesia and were sacrificed. Liver, lungs, spleen, colon, cecum, and blood plasma were snap frozen in liquid nitrogen and stored at −80°C.

### Isolation of Human Peripheral Blood Mononuclear Cells (PBMC), CD3^+^ T-Cells, and Monocyte-Depleted PBMC

For isolation of PBMC, heparinized blood was taken from healthy donors. This procedure and the respective consent documents were approved by the “Ethik Kommission” of the University Hospital Goethe-University Frankfurt. PBMC were isolated from peripheral blood using Histopaque-1077 (Sigma-Aldrich) according to the manufacturer’s instructions. Untouched CD3^+^ T-cell were isolated from PBMC using the Pan-T-cell isolation kit according to the manufacturer’s instructions (Miltenyi, Bergisch Gladbach, Germany). Mean purity was 96.0 ± 0.7% (*n* = 37) determined by FACS analysis using anti-CD3-PerCP/Cy5.5-#UCHT1 (BioLegend). Monocyte-depleted PBMC were generated using anti-CD14 beads (Miltenyi) with a mean depletion efficiency of 98.1 ± 0.7% (*n* = 7) as assessed by FACS analysis using an anti-CD14eFluor450-#61D3 antibody (eBioscience, Frankfurt, Germany). Cells were resuspended in RPMI 1640 supplemented with 10 mM HEPES, 100 U/ml penicillin, 100 µg/ml streptomycin, and 1% human serum (Life Technologies, Darmstadt, Germany) and seeded at 3 × 10^6^ cells/ml in round-bottom polypropylene tubes (Greiner, Frickenhausen, Germany).

### Isolation of Murine Splenocytes

Spleens obtained from 8- to 12-week-old male C57Bl/6 mice (MFD-Diagnostics GmbH) were excised and transferred to 5 ml ice-cold RPMI 1640 medium without FCS. Tissue was destroyed over a nylon cell strainer (70 µm; BD Biosciences, Heidelberg, Germany). Cell suspensions were centrifuged at 500 *g* for 5 min at 4°C and resuspended in 2 ml 0.83% NH_4_Cl for 2 min at RT. Red blood cell lysis was stopped by adding 10 ml cold RPMI 1640 medium without FCS. Splenocytes were collected by centrifugation, washed once with RPMI and resuspended in RPMI 1640, supplemented with 10% heat-inactivated FCS and 100 U/ml penicillin, 100 µg/ml streptomycin. Cells were seeded on 24 well polystyrene plates (Greiner) with 0.5 ml media in a concentration 6 × 10^6^ cells/ml.

### Cultivation of Human Jurkat T Cells, DLD1 and Caco2 Colon Carcinoma Cells, and HepG2 Hepatoma Cells

Jurkat T cells (ATCC-TIB-152) were obtained from the American Type Culture Collection (Manassas, VA, USA) and cultured in RPMI 1640 (Life Technologies) supplemented with 100 U/ml penicillin, 100 µg/ml streptomycin, and 10% heat-inactivated FCS (Life Technologies). For experiments, cells were seeded on 6-well polystyrene plates (Greiner) at a density of 2.5 × 10^6^ cells/ml. DLD1 colon epithelial/carcinoma cells (Center of Applied Microbiology and Research, Salisbury, UK), Caco2 colon epithelial/carcinoma cells, and HepG2 hepatoma cells (German Collection of Microorganisms and Cell Cultures, Braunschweig, Germany) were maintained in DMEM supplemented with 100 U/ml penicillin, 100 µg/ml streptomycin, and 10% heat-inactivated FCS (Life Technologies). For experiments, cells were plated on 6-well polystyrene plates (Greiner) and used in subconfluent condition.

According to the protocols indicated in the figure legends, cells (PBMC, epithelial-like cell lines) were cultivated in parallel at 30, 33, or 37°C incubator temperature. Incubators used for different temperatures were switched occasionally in order to exclude incubator effects on cell behavior different from incubator temperature.

### Cytokine Analysis by Enzyme-Linked Immunosorbent Assay (ELISA)

Murine and human IL-22 (R&D-Systems, Wiesbaden, Germany) and human IL-8 (BD Biosciences) secretion were determined by ELISA according to the manufacturer’s instructions.

### Intracellular Cytokine Staining and Flow Cytometry

Peripheral blood mononuclear cells were kept as unstimulated control or stimulated for 7 h with agonistic anti-CD3 (0.2 µg/ml)/-CD28 (0.02 µg/ml) antibodies at 37 or 33°C. Thereafter, brefeldin A (2 µg/ml) was added for another 4 h, followed by intracellular staining and flow cytometry. After harvesting, PBMC were reconstituted in FACS buffer (1 × PBS + 1% FCS) and stained with surface marker antibody (anti-CD4-PE-Cy7-#SK3, eBioscience) for 30 min on ice. Thereafter, PBMC were fixed and permeabilized [BD Cytofix/Cytoperm Kit (BD Biosciences)], followed by resuspension in FACS buffer, and intracellular staining (2 h on ice) using IL-22-PE-#22URTI or IFNγ-FITC-B27 (both eBioscience) and flow cytometry with gates set to exclude cell debris.

### Analysis of mRNA Expression by Real-time Polymerase Chain Reaction (PCR)

**Figure 1:**
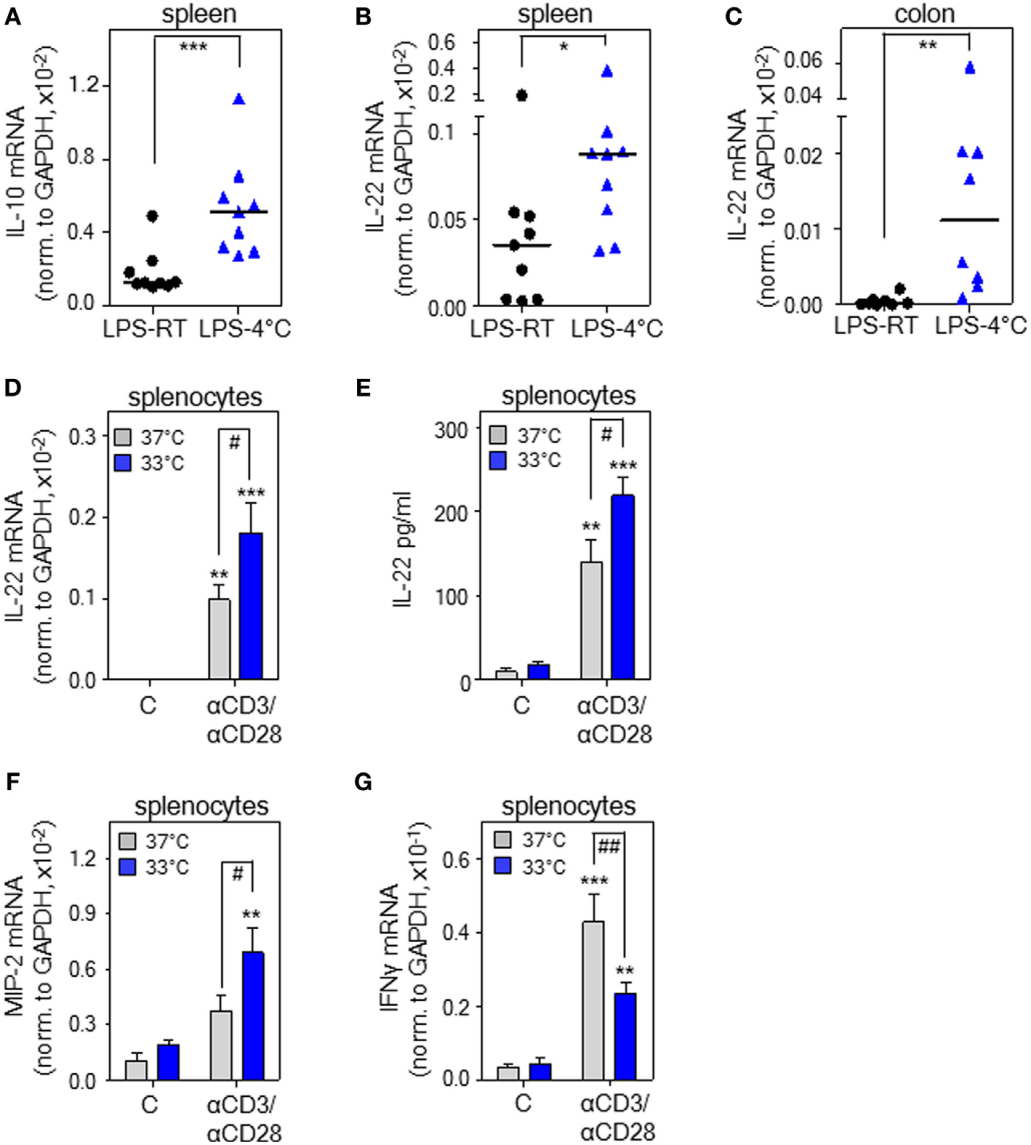
Cold stress and hypothermia promote interleukin (IL)-22 expression as detected in endotoxemic mice and cultured murine splenocytes. **(A–C)** Mice received i.p. either PBS or lipopolysaccharide (LPS, 1 µg/g) under conditions of room temperature (RT) [LPS-RT, *n* = 9 for **(A,B)** and *n* = 8 for **(C)**] or at 4°C [LPS-4°C, *n* = 9 for **(A,B)** and *n* = 8 for **(C)**]. After 5 h, splenic IL-10 **(A)** and IL-22 **(B)** as well as colonic **(C)** IL-22 mRNA expression were determined by real-time polymerase chain reaction (PCR) using GAPDH for normalization. Depicted are raw data for each tissue sample together with the group median. Expression of IL-22 mRNA in PBS-treated control mice (spleen: RT or 4°C, *n* = 8; colon: RT or 4°C, *n* = 6) was undetectable (spleen) or below 0.07 × 10^–5^ (colon) (raw data, IL-22 normalized to GAPDH). **(A–C)** *P < 0.05, **P < 0.01, ***P < 0.001; statistical analysis, Mann–Whitney *U*-test. **(D,F,G)** *Ex vivo* cultured murine splenocytes were either kept as unstimulated control or stimulated with agonistic anti-CD3 (0.2 µg/ml)/-CD28 (0.02 µg/ml) antibodies at 37 or 33°C. After 8 h, mRNA expression of IL-22 (*n* = 10) **(D)**, macrophage inflammatory protein (MIP)-2 (*n* = 5) **(F)**, and interferon (IFN)γ (*n* = 10) **(G)** was determined by real-time PCR using GAPDH for normalization. Depicted are raw data (means ± SEM). **(E)** Murine splenocytes were either kept as unstimulated control or stimulated with agonistic anti-CD3 (5 µg/ml)/-CD28 (0.5 µg/ml) antibodies at either 37 or 33°C. After 72 h, IL-22 secretion was determined by enzyme-linked immunosorbent assay. Data are shown as means ± SEM (unstimulated control at either temperature, *n* = 4; anti-CD3/-CD28 at either temperature, *n* = 5). **(D–G)** **P < 0.01, ***P < 0.001 compared to unstimulated control; ^#^*P* < 0.05, ^##^*P* < 0.01; statistical analysis, one-way ANOVA with *post hoc* Bonferroni correction.

**Figure 2:**
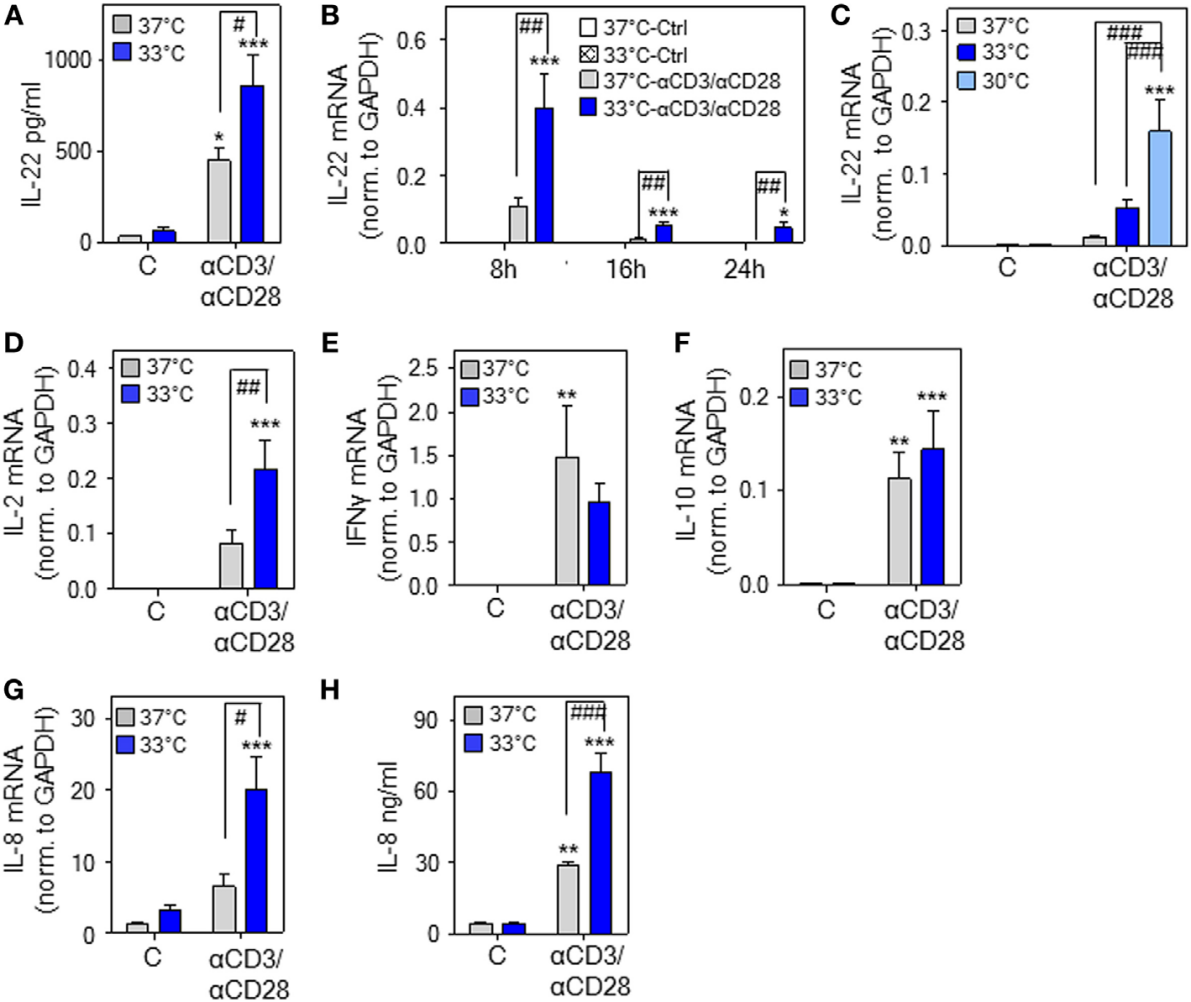
Hypothermia enhances interleukin (IL)-22 expression and release by human peripheral blood mononuclear cells (PBMC). **(A–H)** PBMC were kept as unstimulated control or stimulated with agonistic anti-CD3 (0.2 µg/ml)/-CD28 (0.02 µg/ml) antibodies at 37 or 33°C **(A–H)** or 30°C **(C)**. **(A)** After 48 h, IL-22 secretion was determined by enzyme-linked immunosorbent assay. Data are shown as means ± SEM (*n* = 6). *P < 0.05, ***P < 0.001 compared to unstimulated control at the respective temperature; ^#^*P* < 0.05; statistical analysis, one-way ANOVA with *post hoc* Bonferroni correction. **(B)** After 8 h (*n* = 13), 16 h (unstimulated control at either temperature, *n* = 8; anti-CD3/-CD28 at either temperature, *n* = 14), or 24 h (unstimulated control at either temperature, *n* = 9; anti-CD3/-CD28 at either temperature, *n* = 14), IL-22 mRNA expression was determined by real-time polymerase chain reaction (PCR) using GAPDH for normalization. Depicted are raw data (means ± SEM). *P < 0.05, ***P < 0.001 compared to unstimulated control at the respective temperature and time point; ^##^*P* < 0.01; statistical analysis for individual time points, one-way ANOVA with *post hoc* Bonferroni correction. **(C)** After 16 h [unstimulated at all temperatures, *n* = 8; anti-CD3/-CD28 at 37°C (*n* = 14), at 33°C (*n* = 14), or at 30°C (*n* = 10)], IL-22 mRNA expression was determined by real-time PCR using GAPDH for normalization. Depicted are raw data (means ± SEM). ***P < 0.001 compared to unstimulated control at the respective temperature; ^###^*P* < 0.001; statistical analysis, one-way ANOVA with *post hoc* Bonferroni correction. **(D–G)** After 8 h, IL-2 [**(D)**, *n* = 12], interferon (IFN)γ [**(E)**, *n* = 12], IL-10 [**(F)**, *n* = 14], and IL-8 [**(G)**, *n* = 14] mRNA expression was determined by real-time PCR using GAPDH for normalization. Depicted are raw data (means ± SEM). **P < 0.01, ***P < 0.001 compared to unstimulated control at the respective temperature; ^#^*P* < 0.05, ^##^*P* < 0.01. **(H)** After 48 h, IL-8 secretion was determined by enzyme-linked immunosorbent assay. Data are shown as means ± SEM (*n* = 6). **P < 0.01, ***P < 0.001 compared to unstimulated control at the respective temperature; ^###^*P* < 0.001. **(D–H)** Statistical analysis, one-way ANOVA with *post hoc* Bonferroni correction.

Total RNA was extracted from homogenized mouse tissue or cultured cells using Tri-Reagent according to the manufacturer’s instructions (Sigma-Aldrich). Tissues were homogenized using OMNI TIP Homogenizing KIT (Kennesaw, GA, USA). 0.5 µg RNA was transcribed using random hexameric primers and Moloney Murine Leukemia Virus Reverse Transcriptase (Thermo Scientific, Darmstadt, Germany) according to the manufacturer’s instructions. cDNA was amplified using assay-on-demand kits (Taqman probes/assay kit from Thermo Scientific) and an AbiPrism 7500 Fast Sequence Detector (Thermo Scientific). During real-time PCR, changes in fluorescence are caused by the Taq polymerase degrading a probe containing a fluorescent dye [glyceraldehyde 3-phosphate dehydrogenase (GAPDH): VIC; all others: FAM]. Two initial steps at 50°C for 2 min and 95°C for 20 s were followed by 40 cycles at 95°C for 3 s and 60°C for 30 s. Target mRNA was normalized to that of GAPDH and quantified by the 2^–ΔCT^ method (raw data, Figures 1, 2, 4 and 5) or the 2^–ΔΔCT^ method (fold-induction, Figure 6). The following probes were used: hs-GAPDH (4310884E), hs-IL-22 (Hs01574152_g1), hs-IL-10 (Hs99999035_m1), hs-IFNγ (Hs00174143_m1), α1ACT (Hs00153674_m1), hs-IL-8 (Hs00174103_m1), hs-IL-2 (Hs00174114_m1), mm-GAPDH (4352339E), mm-IL-22 (Mm00444241_m1), mm-MIP2 (Mm00436450_m1), mm-IL-10 (Mm00439614_m1), mm-TNF-α (Mm00443285_m1), mm-IFNγ (Mm01168134_m1), and mm-IL17A (Mm00439618_m1). Primers and probe for IL-18BPa were designed using Primer Express (Applied Biosystems) according to AF110798: forward, 5′-ACCTCCCAGGCCGACTG-3′; reverse, 5′-CCTTGCACAGCTGCGTACC-3′; probe 5′-CACCAGCCGGGAACGTGGGA-3′. GAPDH was not a target of regulation by hypothermia under all conditions investigated (data not shown).

### Immunoblot Analysis

Immunoblot analysis for cellular STAT1/3 in DLD1, Caco2, and HepG2 cells was performed as previously described (37) using total cell lysis buffer [150 mM NaCl, 1 mM CaCl_2_, 25 mM Tris–Cl (pH 7.4), 1% Triton X-100] supplemented with protease inhibitor cocktail (Roche Diagnostics, Mannheim, Germany), DTT, Na_3_VO_4_, PMSF (each 1 mM), and NaF (20 mM). Antibodies: total STAT3-#124H6 (mouse monoclonal antibody); total STAT1, pSTAT1-Y701-#D4A7 (both rabbit polyclonal antibodies); pSTAT3-Y705-#D3A7 (rabbit monoclonal antibody); all from Cell Signaling, Frankfurt, Germany. For detection of total STAT1 or total STAT3 blots were stripped and reprobed.

Isolation of nuclei (38) for immunoblot analysis of nuclear NFAT-c2 in human T-cells was performed by lysis using nuclear extraction buffer A (10 mM HEPES at pH 7.9, 10 mM KCL, 0.1 mM EDTA, 0.1 mM EGTA) supplemented with protease inhibitor cocktail (Roche Diagnostics). After 10 min on ice and addition of Triton X-100, nuclei were collected by centrifugation at 12,000 *g* for 1 min at 4°C. Pellets containing nuclei were resuspended in nucleic-lysis buffer C (20 mM HEPES at pH 7.9, 0.4 M NaCl, 1 mM EDTA, 1 mM EGTA, 25% glycerin) supplemented with protease inhibitor cocktail (Roche Diagnostics). Antibodies: NFAT-c2-#4G6-G5, mouse monoclonal antibody (Santa Cruz Biotechnology, Heidelberg, Germany); β-actin-#AC15, mouse monoclonal antibody (Sigma-Aldrich). For detection of NFAT-c2 and β-actin on the same blot, the blot was cut. Data quantifications were performed by Quantity-One analysis software (Bio-Rad, Munich, Germany).

### Statistical Analysis

Data are shown as group median, means ± SD, or means ± SEM and presented as [raw data], [fold-induction], [percent], [pg/ml], [ng/ml], and [Adj.Vol. INT*mm2]. The D’Agostino–Pearson normality test was used to assess data distribution. Statistical analysis was performed on raw data as indicated in the legends by one-way ANOVA with *post hoc* Bonferroni correction, unpaired Student’s *t*-test, or Mann–Whitney *U*-test. Differences were considered significant in case of *P* values below 0.05 (Prism 5.0, GraphPad, La Jolla, CA, USA).

## Results

### Cold Stress and Hypothermia Promote *il22* Gene Expression As Detected in Endotoxemic Mice and Cultured Splenocytes

Experimental endotoxemia is regarded a standard model for the hyper-inflammatory phase of sepsis. Both, rodent endotoxemia (39) and sepsis (40) associate with enhanced IL-22 production. To investigate *il22* gene expression under the influence of cold stress, PBS-treated control mice and mice undergoing endotoxemia were exposed to either an ambient temperature of 4°C or to RT (23°C) for 5 h. Notably, a low endotoxin dosage of 1 µg/g, by itself unable to induce hypothermia, was administered for induction of systemic inflammation. Analysis of rectal temperature after 5 h at 4°C revealed that only endotoxemic mice (*n* = 9) but not PBS-treated control mice (*n* = 8) developed mild-to-moderate hypothermia with a significant drop from 35.9± 0.3°C to a core temperature of 30.9 ± 0.9°C (*P* < 0.001, Student’s *t*-test). Under conditions of RT, core body temperature of PBS-treated control (*n* = 8) or endotoxemic mice (*n* = 9) was indistinguishable at 35.8 ± 0.4 or 35.5 ± 0.4°C, respectively. In accord with previous *in vivo* data on IL-10 and anti-inflammatory properties of hypothermia (26–35), enhanced splenic *il10* gene expression was observed in endotoxemic mice exposed to 4°C (Figure 1A). To verify the hypothesis that IL-22 is affected by hypothermia, these same specimens were analyzed for expression of this cytokine. As shown in Figure 1B, splenic *il22* gene expression was significantly upregulated in endotoxemic mice exposed to 4°C ambient temperature. On the contrary, in endotoxemic mice, upregulation of splenic IL-17A mRNA [0.40 × 10^–4^ versus 0.42 × 10^–4^ (median of target gene expression normalized to GAPDH) for RT versus 4°C ambient temperature (*n* = 9), not significantly different by Mann–Whitney *U*-test; IL-17A mRNA, undetectable in PBS-treated control mice—irrespective of ambient temperature] and tumor necrosis factor (TNF)-α mRNA [6.7 ± 0.9- versus 6.4 ± 1.7-fold-induction for RT (*n* = 8) versus 4°C ambient temperature (*n* = 9)] was unaffected by changes in ambient temperature. Since local IL-22 is crucial for maintenance of intestinal barrier function (41) and sepsis pathogenesis (16), colonic IL-22 expression was determined and found likewise upregulated upon cold stress (Figure 1C). Induction of colonic TNF-α mRNA by endotoxemia was, in accord with observations in spleen, not further increased by changes in ambient temperature (data not shown). Notably, as compared to RT, an ambient temperature of 4°C without endotoxemia failed to significantly affect splenic and colonic *il22* expression. Specifically, using the current protocol, IL-22 mRNA was neither detectable in total colonic RNA obtained from PBS-treated control mice exposed to RT (*n* = 6) nor in splenic specimens irrespective of the tested ambient temperature (*n* = 8). In colonic tissues obtained from PBS-treated control mice exposed to 4°C, *il22* expression was very low and barely detectable with a median of 0.01 × 10^–5^ (*n* = 6) for IL-22 mRNA normalized to that of GAPDH. Notably, very low *il22* gene expression in colonic tissue of healthy untreated mice concurs with previous observations (42).

Exposure of mice to cold stress engages a complex systemic response that involves, among others, activation of the β-adrenergic/cAMP-axis (20) with its documented potential for immunomodulation (43), possibly by upregulation of IL-10 (43, 44). In order to evaluate whether hypothermia is able to directly upregulate *il22* expression on the level of cultured murine leukocytes, freshly isolated splenocytes were stimulated using agonistic anti-CD3/-CD28 antibodies in the context of an ambient temperature of either 37 or 33°C—the latter condition resembling mild hypothermia. Figure 1D demonstrates that mild hypothermia amplifies IL-22 mRNA expression by activated splenocytes. Moreover, we also verified the potential of hypothermia to upregulate IL-22 protein release (Figure 1E). Whereas the murine functional IL-8 homolog and stress-responsive parameter macrophage inflammatory protein (MIP)-2 displayed similar upregulation by hypothermia (Figure 1F), expression of IFNγ mRNA was retarded under these same conditions (Figure 1G). In light of the proinflammatory pathological functions of IFNγ (45), this latter observation agrees with immunosuppressive properties of hypothermia (46). Taken together, data suggest that cold stress and hypothermia can serve as a cofactor enhancing murine *il22* gene expression. Since stimulatory effects of hypothermia on IL-22 are detectable on cell culture level (Figures 1D,E), upregulation of the cytokine *in vivo* may also be independent from activation of the β-adrenergic/cAMP-axis.

### Hypothermia Enhances IL-22 Expression and Release by Human PBMC

To broaden aforementioned murine *in vitro* and *in vivo* data, experiments were performed using human PBMC. We confirm previous observations (47) on IL-22 secretion by PBMC in response to stimulatory anti-CD3/-CD28 antibodies at physiological 37°C. Herein, we demonstrate that cultivation of PBMC at 33°C significantly amplifies IL-22 protein secretion (Figure 2A). Enhanced expression of IL-22 under the influence of mild hypothermia and anti-CD3/-CD28-stimulation was likewise detectable on mRNA level (Figure 2B). Upregulation of IL-22 by hypothermia was even more pronounced by further reducing the cultivation temperature to 30°C (Figure 2C) indicating a concurrent response upon more severe hypothermic culture conditions. *IL2* gene expression was, like *IL22*, enhanced upon cultivation at 33°C (Figure 2D). Notably, regulation by mild hypothermia displayed specificity since neither expression of *IFNG* (Figure 2E) nor that of *IL10* (Figure 2F) was affected by cultivation of anti-CD3/-CD28-stimulated PBMC at 33°C. Unresponsiveness of IL-10 concerning hypothermia *in vitro* contrasts with robust IL-10 upregulation *in vivo* [(28–35) and Figure 1A]. IL-10 production *in vivo* is, however, induced by the β-adrenergic/cAMP-axis (44), which is activated by acute hypothermia (20). This likely explains the observed differences compared to *in vitro* cultured PBMC. In contrast to IFNγ and IL-10, mRNA expression (Figure 2G) and release (Figure 2H) of IL-8 was increased by mild hypothermia. This latter observation concurs with the notion that *IL8* is a stress-responsive gene (48).

**Figure 3:**
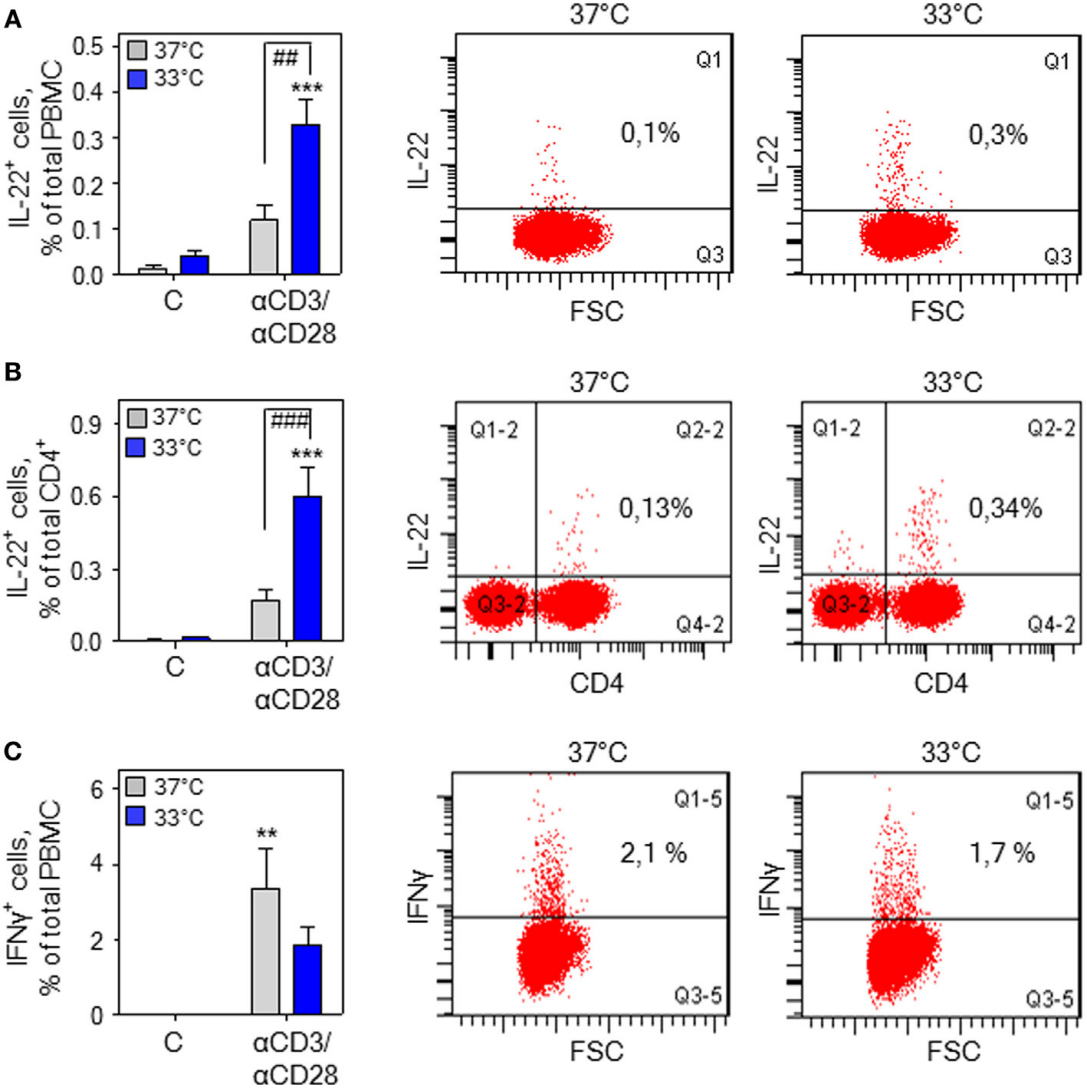
Hypothermia increases interleukin (IL)-22^+^ cells in total peripheral blood mononuclear cells (PBMC). **(A)** PBMC were kept as unstimulated control or stimulated for 7 h with agonistic anti-CD3 (0.2 µg/ml)/-CD28 (0.02 µg/ml) antibodies at 37 or 33°C. Thereafter, brefeldin A (2 µg/ml) was added for another 4 h, followed by intracellular staining and flow cytometry for IL-22 **(A,B)** and interferon (IFN)γ **(C)** detection. Left panel **(A–C)**: depicted are percentages of IL-22^+^ cells in total PBMC (*n* = 6) **(A)** and the CD4^+^ PBMC fraction (*n* = 7) **(B)** as well as IFNγ^+^ cells in total PBMC (*n* = 6) **(C)**. Data are shown as means ± SEM. **P < 0.01, ***P < 0.001 compared to unstimulated control at the respective temperature; ^##^*P* < 0.01, ^###^*P* < 0.001; statistical analysis, one-way ANOVA with *post hoc* Bonferroni correction. Right panel: depicted are representative dot blots displaying IL-22^+^ cells in total PBMC **(A)** and the CD4^+^ PBMC fraction **(B)** as well as IFNγ^+^ cells in total PBMC **(C)**.

Stimulation of human PBMC by treatment with anti-CD3/-CD28 antibodies and associated IL-22 secretion has been linked to the CD4^+^ memory T-cell compartment being the chief IL-22 PBMC-source under conditions of polyclonal activation (47). Herein, intracellular cytokine staining demonstrated that upregulation of IL-22^+^ cells by anti-CD3/-CD28 in total PBMC (Figure 3A) and the CD4^+^ PBMC fraction (Figure 3B) is further amplified by mild hypothermia. In contrast, exposure to 33°C did not significantly affect numbers of IFNγ^+^ cells in total PBMC (Figure 3C) thus confirming aforementioned mRNA data (Figure 2E).

### Hypothermia Amplifying *IL22* Expression in PBMC Demands the Presence of Monocytes

**Figure 4:**
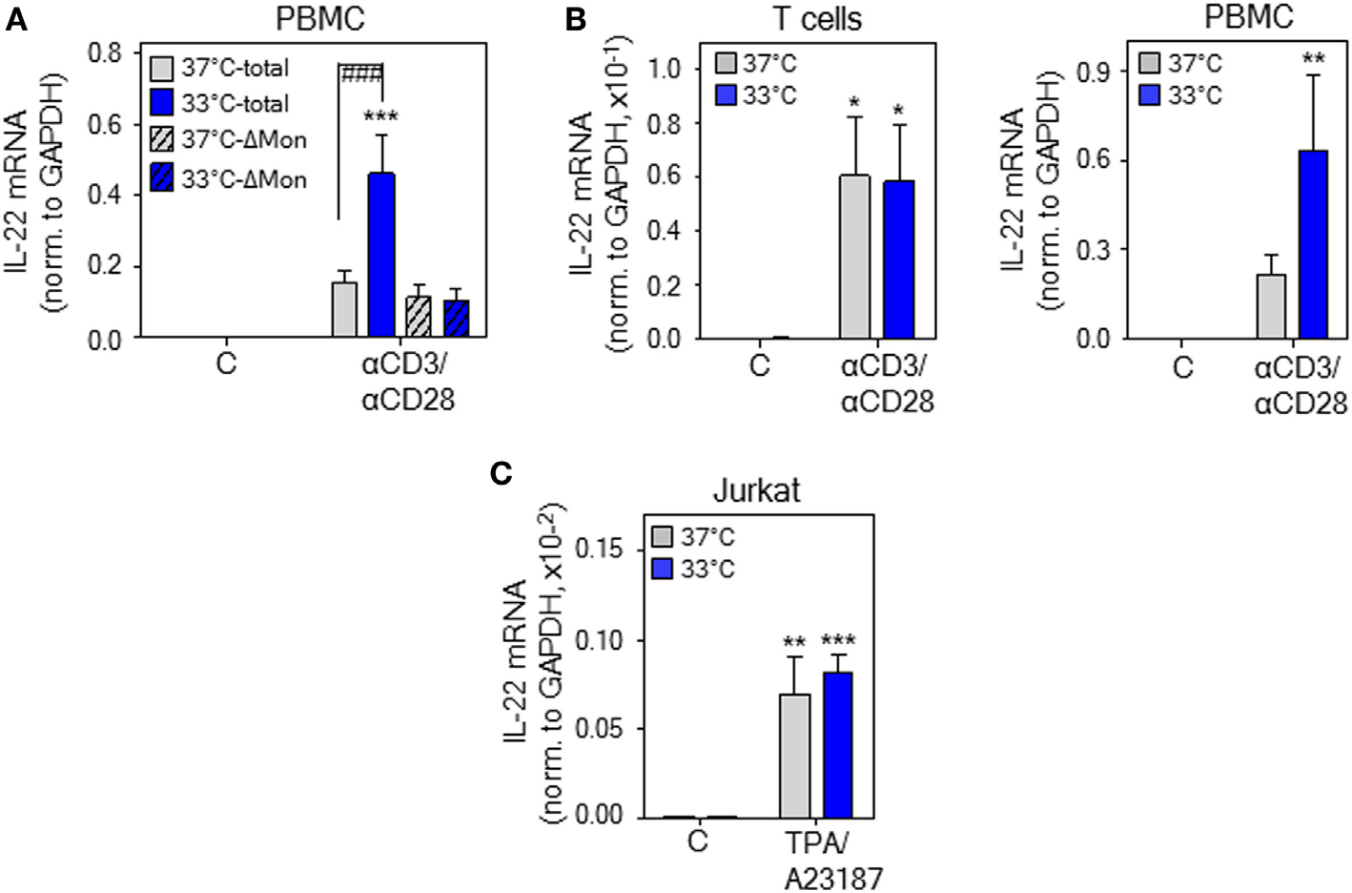
Upregulation of interleukin (IL)-22 expression by hypothermia demands monocyte presence. **(A)** Peripheral blood mononuclear cells (PBMC) were used in parallel as whole population (total) or depleted for monocytes (ΔMon). Total and monocyte-depleted PBMC were kept as unstimulated control or stimulated with anti-CD3 (0.2 µg/ml)/-CD28 (0.02 µg/ml) antibodies at 37 or 33°C. After 8 h, IL-22 mRNA expression was determined by real-time polymerase chain reaction (PCR) using GAPDH for normalization. Data are shown as means ± SEM (*n* = 7). **(B)** PBMC were used in parallel as whole population or as starting point for isolation of CD3^+^ T-cells. CD3^+^ T-cells (left panel) or total PBMC (right panel) were kept as unstimulated control or stimulated with anti-CD3 (0.2 µg/ml)/-CD28 (0.02 µg/ml) antibodies at 37 or 33°C. After 8 h, IL-22 mRNA expression was determined by real-time PCR using GAPDH for normalization. Data are shown as means ± SEM (*n* = 9). **(C)** Jurkat T cells were kept as unstimulated control or stimulated with TPA (20 ng/ml) plus A23187 (2 µM) at 37 or 33°C. After 6 h, IL-22 mRNA expression was assessed by real-time PCR using GAPDH for normalization. Data are shown as means ± SD (*n* = 5). **(A–C)**, *P < 0.05, **P < 0.01, ***P < 0.001 compared to unstimulated control at the respective temperature; ^###^*P* < 0.001; statistical analysis, one-way ANOVA with *post hoc* Bonferroni correction.

Monocytes have previously been identified as crucial cellular component supporting T-cell-derived IL-22 production by PBMC in response to diverse stimuli (49, 50). To specify their role in mild hypothermia enhancing IL-22 production, PBMC were depleted from monocytes. As shown in Figure 4A, depletion of monocytes entirely prevented upregulation of *IL22* expression under the influence of hypothermia. To further investigate this matter, whole T-cells were isolated from PBMC and cultivated thereafter at either 37 or 33°C. Analysis after stimulation of isolated T-cells by anti-CD3/-CD28 antibodies actually revealed no significant difference in *IL22* expression between both ambient temperatures (Figure 4B, left panel). Notably, whole PBMC from these same donors simultaneously cultivated displayed amplified IL-22 mRNA expression at 33°C (Figure 4B, right panel). Human leukemic Jurkat T-cells activate *IL22* gene expression in response to polyclonal stimulation (18). In accord with aforementioned data on isolated primary T-cells, mild hypothermia left *IL22* expression by TPA/A23187-stimulated Jurkat T-cells unaffected (Figure 4C). Taken together, observations indicate that upregulation of IL-22 by mild hypothermia is not a T-cell autonomous process but requires interactions between T cells and additional PBMC subsets, especially monocytes.

### Hypothermia Promotes NFAT-c2 in Human PBMC

**Figure 5:**
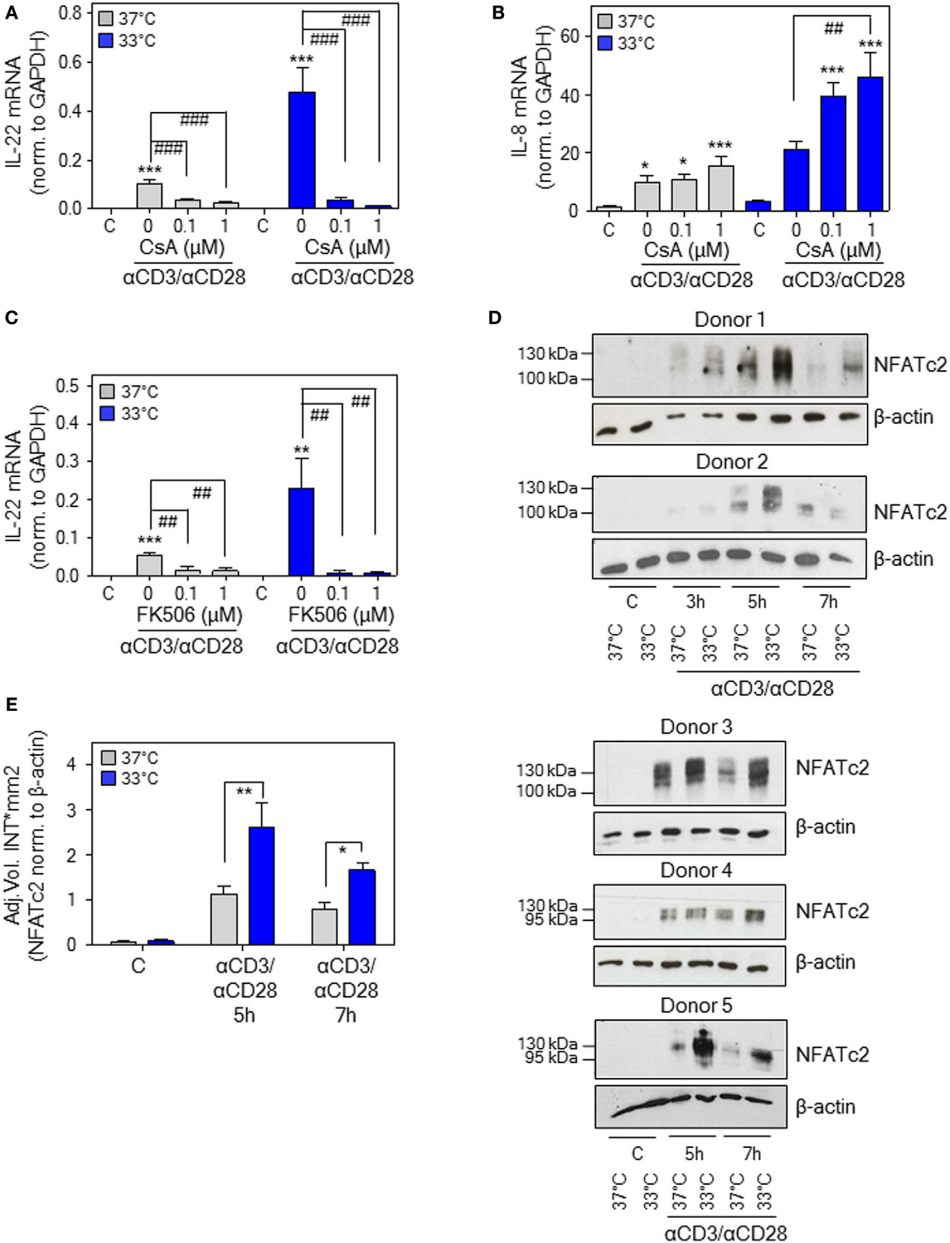
Nuclear factor of activated T cells (NFAT) signaling in peripheral blood mononuclear cells (PBMC) is mandatory for interleukin (IL)-22 upregulation by anti-CD3/-CD28 upon normothermic or hypothermic culture conditions. **(A–C)** Where indicated, PBMC were pretreated for 30 min with CsA **(A,B)** or with FK506 **(C)** at the specified concentrations. Cells were further kept as unstimulated control or stimulated with anti-CD3 (0.2 µg/ml)/-CD28 (0.02 µg/ml) antibodies at 37 or 33°C. All cultures were adjusted to a final concentration of 0.002% **(A,B)** or 0.008% DMSO **(C)** (vehicle for CsA and FK506). After 8 h, IL-22 [**(A)**, *n* = 10; **(C)**, *n* = 6] or IL-8 [**(B)**, *n* = 10] mRNA expression was assessed by real-time polymerase chain reaction using GAPDH for normalization. Data are shown as means ± SEM. *P < 0.05, **P < 0.01, ***P < 0.001 compared to unstimulated control; ^##^*P* < 0.01, ^###^*P* < 0.001; statistical analysis for either 37 or 33°C, one-way ANOVA with *post hoc* Bonferroni correction. **(D)** PBMC from five different donors were kept unstimulated or stimulated with anti-CD3 (0.2 µg/ml)/-CD28 (0.02 µg/ml) antibodies at 37 or 33°C for the indicated time periods. Thereafter, CD3^+^ T-cell isolation was performed followed by immunoblot analysis for detection of nucleic NFAT-c2. **(E)** Densitometric quantification of experiments shown in Figure 5D. Data are expressed as means ± SEM. *P < 0.05, **P < 0.01; statistical analysis, unpaired Student’s *t*-test.

The calcineurin/NFAT inhibitors cyclosporin A (CsA) and FK506 (tacrolimus) (51) potently suppress *IL22* expression by activated human Jurkat T-cells, primary T-cells, and PBMC (18, 52). This observation concurs with inhibition of T-cell-derived IL-22 in psoriatic skin of tacrolimus-treated mice (53) and downregulation of IL-22 in patients undergoing CsA therapy (54, 55). Moreover, NFAT-c2 (51) binding to a specific site within the human *IL22* promoter contributes to gene activation (18), conceivably by cooperation with an adjacent binding site for ATF2-jun heterodimers (56). Since aforementioned data suggested IL-22 as NFAT-inducible cytokine, CsA was tested in the current experimental protocol. In fact, coincubation with CsA not only impaired *IL22* expression by activated PBMC upon normothermia but entirely prevented potentiation of gene induction at 33°C (Figure 5A). In contrast, CsA failed to suppress anti-CD3/-CD28-induced IL-8, a monocyte-derived inflammatory chemokine that was determined to control for unspecific inhibitory effects of the agent on PBMC cytokine expression. Interestingly, CsA actually amplified *IL8* expression upon hypothermia (Figure 5B), which corresponds to observations on human smooth muscle cells where stimulation of activator protein-1 by CsA enforces IL-8 (57). Notably, the alternate NFAT antagonist FK506 (tacrolimus) displayed very similar inhibitory action on IL-22 (Figure 5C). As expected, we confirm previous observations (58) on potent suppression of IFNγ production by PBMC under the influence of CsA (Figure S1 in Supplementary Material).

In order to more directly investigate the role of NFAT-c2 in this context, immunoblot analysis was performed using T-cells isolated instantly after stimulation of PBMC at 33 or 37°C. Herein, we demonstrate that hypothermia increases nuclear NFAT-c2 accumulation, thus transcription factor activation, in primary human T-cells after activation by anti-CD3/-CD28 (as part of whole PBMC). Notably, results obtained from five different donors indicated the most prominent effect of low ambient temperature at 5–7 h after onset of polyclonal T-cell stimulation (Figure 5D). Densitometric quantification of these experiments is shown in Figure 5E. Data altogether suggest that exposure of PBMC to mild hypothermia in CsA/FK506-sensitive manner supports *IL22* expression, which associates with and is likely mediated by enhanced nuclear NFAT-c2.

### Hypothermia Supports Activation of Epithelial-Like Cells by IL-22

**Figure 6:**
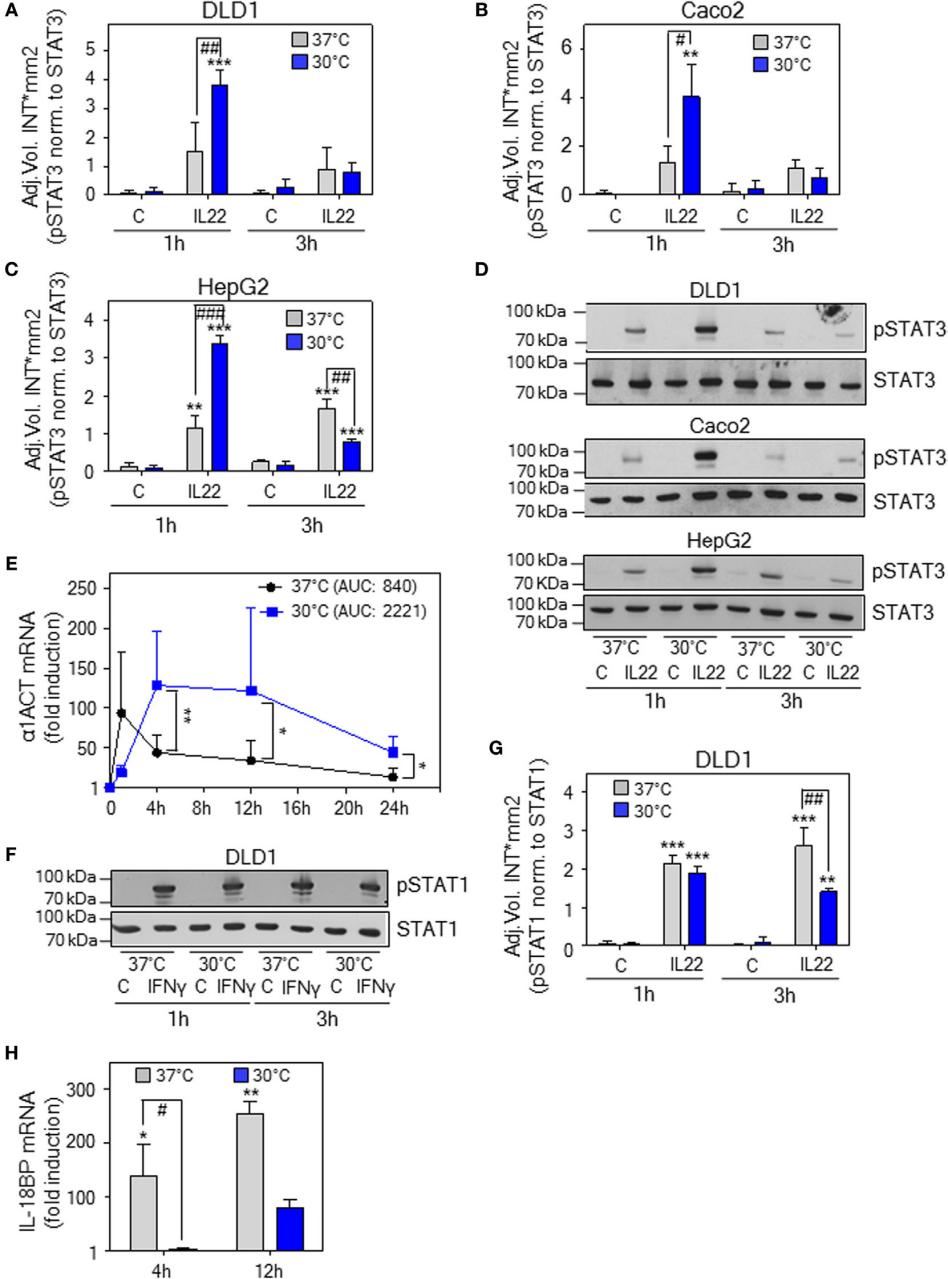
Effects of hypothermic preconditioning on interleukin (IL)-22 bioactivity. Epithelial (-like) IL-22-responsive human DLD1 **(A)** and Caco2 colon **(B)** carcinoma cells as well as HepG2 hepatoma cells **(C)** were preincubated for 6 h without additional stimulation at 30 or 37°C. Thereafter, cells maintained under the respective ambient temperatures were further kept as unstimulated control or stimulated with IL-22 (20 ng/ml). After 1 or 3 h, cells were harvested and IL-22-induced signal transducer and activator of transcription (STAT)-3 activation was determined by immunoblot analysis for pSTAT3. **(A–C)** Densitometric quantification of experiments (*n* = 3) is depicted as raw data (means ± SD). **P < 0.01, ***P < 0.001 compared to unstimulated control at the respective temperature and time point; ^#^*P* < 0.05, ^##^*P* < 0.01, ^###^*P* < 0.001; statistical analysis for individual time points, one-way ANOVA with *post hoc* Bonferroni correction. **(D)** One representative of the three independently performed experiments (for each cell type) is shown. **(E–H)** DLD1 cells were preincubated for 6 h without additional stimulation at 30 or 37°C. **(E)** Thereafter, cells were further kept under the respective ambient temperatures as unstimulated control or were stimulated with IL-22 (20 ng/ml). After the indicated time periods, α1ACT mRNA expression was determined by real-time polymerase chain reaction (PCR) using GAPDH for normalization. Data are shown as means ± SD [1 h, *n* = 5 (30 and 37°C); 4 h, *n* = 8 (30°C) and *n* = 7 (37°C); 12 h, *n* = 8 (30 and 37°C); 24 h, *n* = 6 (30°C), and *n* = 4 (37°C)]; *P < 0.05, **P < 0.01; statistical analysis on fold-induction at each time point, unpaired Student’s *t*-test. AUC, area under the curve. **(F)** Thereafter, cells were further kept under the respective ambient temperatures as unstimulated control or stimulated with interferon (IFN)γ (20 ng/ml). After 1 or 3 h, cells were harvested and IFNγ-induced STAT1 activation was determined by immunoblot analysis for pSTAT1. One representative of three independently performed experiments is shown. Densitometric quantification of these experiments (*n* = 3) is depicted in **(G)** as raw data (means ± SD). **P < 0.01, ***P < 0.001 compared to unstimulated control at the respective temperature and time point; ^##^*P* < 0.01; statistical analysis for individual time points, one-way ANOVA with *post hoc* Bonferroni correction. **(H)** Thereafter, cells were further kept under the respective ambient temperatures as unstimulated control or stimulated with IFNγ (20 ng/ml). After 4 or 12 h, IL-18 binding protein (IL-18BP) mRNA expression was determined by real-time PCR using GAPDH for normalization. Data are shown as means ± SD (4 h, *n* = 4; 12 h, *n* = 5); *P < 0.05, **P < 0.01 compared to unstimulated control of the respective temperature and time point; ^#^*P* < 0.05; statistical analysis on raw data for individual time points, one-way ANOVA with *post hoc* Bonferroni correction.

To investigate effects of hypothermia on IL-22 biological activity, IL-22-responsive epithelial-like human Caco2 and DLD1 colon carcinoma cells (59) as well as HepG2 hepatoma cells (13) were adjusted for 6 h to an ambient temperature of either 30 or 37°C followed by stimulation with IL-22. As detected by analysis of pSTAT3 after 1 h, early IL-22 signal transduction was significantly enhanced upon hypothermia in all three cell types investigated. Notably, this effect vanished at the later 3-h time point. Figure 6 displays densitometric quantification of results (three independently performed experiments per cell line) obtained from DLD1 (Figure 6A), Caco2 (Figure 6B), and HepG2 cells (Figure 6C). Representative immunoblots for each cell line are shown in Figure 6D. To determine consequences of temperature-sensitive STAT3 activation for downstream gene regulation, prototypic IL-22-inducible α1-antichymotrypsin (α1ACT) (1, 14) was investigated in DLD1 cells. Time course analysis revealed enhanced expression of α1ACT mRNA at 30°C, a phenomenon that started at 4 h and persisted thereafter (Figure 6E). The specificity of hypothermia supporting STAT3 was assessed by analysis of IFNγ-mediated activation of STAT1 using the same experimental protocol. As shown by immunoblot analysis (Figure 6F) and respective densitometric quantification of three independently performed experiments (Figure 6G), STAT1 activation was, in stark contrast to STAT3, not enhanced but moderately inhibited by exposure of DLD1 cells to hypothermia. Moreover, expression of prototypic STAT1-dependent IL-18 binding protein (IL-18BP) mRNA expression (60, 61) was curbed by cultivation at 30°C (Figure 6H).

## Discussion

Herein, we identify *in vitro* and *in vivo* hypothermia as novel parameter augmenting IL-22 expression in the context of immunoactivation. Hypothermia as single stimulus, however, failed to significantly upregulate IL-22 under all conditions investigated. Fine-tuning by hypothermia of largely T-cell-derived IL-22 in activated PBMC was dependent on monocytes, sensitive to inhibition by CsA or FK506, and associated with increased nuclear NFAT-c2. The notion of enhanced NFAT-c2 function under the influence of hypothermia is furthermore supported by the current observation of similarly regulated IL-2 expression, a well-defined prototypic NFAT-c2-inducible gene (62). Notably, early data already indicated the capability of monocytes to amplify Ca^2+^-signaling, thus NFAT function, in adjacent T-cells (63). Since NFAT in T-cells can be engaged by T-cell receptor-independent mechanisms connecting to innate immunity (64, 65), enhanced NFAT-c2 may also contribute to the current observation of hypothermia-associated IL-22 upregulation during murine endotoxemia.

Hypothermia may support NFAT function by action on T cell receptor/Ca^2+^-signaling and calcineurin activation or by inducing a state of NFAT hypophosphorylation *via* suppression of deactivating kinases. Interestingly, inhibition of glycogen synthase kinase-3β, regarded key to NFAT inactivation (51), has been associated with hypothermia in rat lung injury (66). In light of increased nuclear NFAT-c2 and enhanced IL-2 as well as IL-22 expression, lack of IFNγ upregulation, likewise an NFAT target (67), indicates complexity of hypothermia-regulated cytokine production. Hypothermia may thus be able to inhibit additional signals that are required for IFNγ production but leave IL-22 unaffected. Whereas inhibition of IFNγ agrees with tissue-protective properties of hypothermia, future studies need to shed light on mechanisms of differential regulation of IFNγ and IL-22 by low ambient temperature.

In order to relate effects of lowered ambient temperature to IL-22 biological activity, STAT3 activation was investigated. Previous reports indicated that activation of hepatic STAT3 is enforced by hypothermia during piglet cardiac surgery (29) and murine liver regeneration (68). Both studies connect hypothermia and hepatic STAT3 activation to liver protection, though upregulation of either IL-10 (29) or IL-6 (68) was associated with those observations—leaving open the possibility that hypothermia directly affects signal transduction. Herein, we report that cultivation of human DLD1 and Caco2 colon carcinoma as well as HepG2 hepatoma cells under the influence of hypothermia amplifies initial IL-22 signal transduction. Data concur with increased STAT3 activation in murine brain endothelial cells exposed to hypothermia (69). Notably, tissue-protective IL-22/STAT3 (4) but decisively not IFNγ/STAT1 signaling, the latter known to support pathological inflammation (45), was enhanced by hypothermia in the current study.

Tissue stress unbalancing homeostasis interconnects in a regulatory network with a fine-tuned inflammatory program, recently coined para-inflammation, which has the potential to enable adaptation and possibly protective preconditioning (70, 71). In that broader context, upregulation of IL-22 by hypothermia in endotoxemic mice suggests this cytokine to be part of a protective agenda not only directly serving preservation of stressed/injured tissues. By its capability to enforce insulin action (72, 73), enhanced IL-22 biological activity may, moreover, support insulin-dependent glucose uptake by brown fat and muscle tissue, which likely contributes to or supports heat generation of the hypothermic organism (74–76). Notably, when studied individually, endotoxemia and hypothermia are generally associated with reduced insulin function (77–80).

Rodent models of severe systemic inflammation/sepsis display endogenous hypothermia (81, 82) that can serve defined protective functions (19, 30–35). Current data suggest upregulation of IL-10 (30–35) and IL-22 as part of a hypothermia-associated cytokine profile counteracting overt pathological inflammation and strengthening biological barriers during severe systemic inflammation and infection. Observations presented likewise imply that IL-22 may contribute to specific tissue-protective properties of elective hypothermia.

## Ethics Statement

For isolation of PBMC, heparinized blood was taken from healthy donors. This procedure was carried out in accordance with the recommendations of “Ethik Kommission of the University Hospital Goethe-University Frankfurt” with written informed consent from all subjects. All subjects gave written informed consent in accordance with the Declaration of Helsinki. The protocol was approved by the “Ethik Kommission of the University Hospital Goethe-University Frankfurt.” *In vivo* mouse experiments were carried out in accordance with the recommendations of ‘Regierungspräsidium Darmstadt’. The protocol was approved by the ‘Regierungspräsidium Darmstadt’.

## Author Contributions

EC, BH, and MB performed experiments, analyzed the data, and performed manuscript editing. JP analyzed the data and contributed to manuscript editing. HM analyzed the data, designed the study, wrote the paper, and performed manuscript editing.

## Conflict of Interest Statement

The authors declare that the research was conducted in the absence of any commercial or financial relationships that could be construed as a potential conflict of interest.
