# Supplementary figures and images for: Hypothermia Promotes Interleukin-22 Expression and Fine-Tunes Its Biological Activity
Source: Front Immunol. 2017 Jun 29;8:742. doi: 10.3389/fimmu.2017.00742 (PMC5489602; doi:10.3389/fimmu.2017.00742)

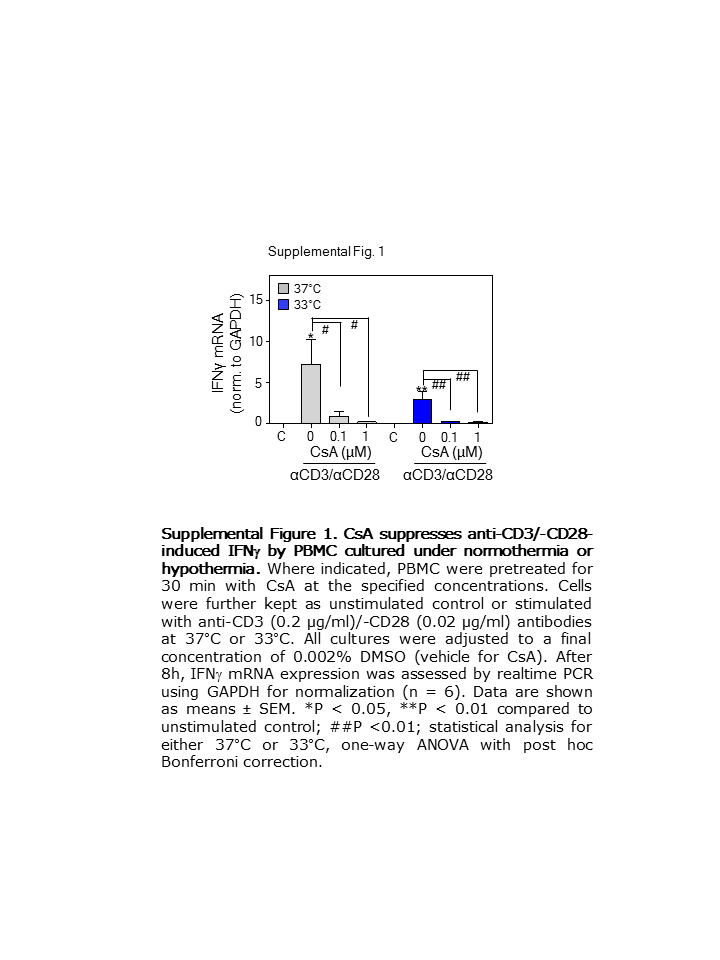

Supplement: Supplementary file 1 [file Image_1.TIF]
